# Phenolic Composition and Antioxidant, Anti-Inflammatory, Cytotoxic, and Antimicrobial Activities of Cardoon Blades at Different Growth Stages

**DOI:** 10.3390/biology11050699

**Published:** 2022-05-02

**Authors:** Filipa Mandim, Spyridon A. Petropoulos, José Pinela, Maria Inês Dias, Marina Kostic, Marina Soković, Isabel C. F. R. Ferreira, Celestino Santos-Buelga, Lillian Barros

**Affiliations:** 1 Centro de Investigação de Montanha (CIMO), Instituto Politécnico de Bragança, Campus de Santa Apolónia, 5300-253 Bragança, Portugal; filipamandim@ipb.pt (F.M.); jpinela@ipb.pt (J.P.); maria.ines@ipb.pt (M.I.D.); iferreira@ipb.pt (I.C.F.R.F.); 2 Grupo de Investigación en Polifenoles (GIP-USAL), Facultad de Farmacia, Universidad de Salamanca, Campus Miguel de Unamuno s/n, 37007 Salamanca, Spain; csb@usal.es; 3 Department of Agriculture, Crop Production and Rural Environment, University of Thessaly, 38446 Volos, Greece; 4 Institute for Biological Research “Siniša Stanković”-National Institute of Republic of Serbia, University of Belgrade, Bulevar Despota Stefana 142, 11000 Belgrade, Serbia; marina.kostic@ibiss.bg.ac.rs (M.K.); mris@ibiss.bg.ac.rs (M.S.)

**Keywords:** cardoon blades, phenolic compounds, phenological growth stage, antioxidant activity, tumor cell growth inhibition, antibacterial/antifungal activity, sustainable ingredients

## Abstract

**Simple Summary:**

The rapid increase of the world population has promoted a more sustainable and efficient use of natural resources. To achieve complete and proper upcycling of plant crops, it is important to know their potential for industrial exploitation. Cardoon (*Cynara cardunculus* L.) is a species native to the Mediterranean basin widely used in different sectors, including food and pharmaceuticals. Despite their multiple industrial applications, not all plant tissues have been incorporated into the value chain. Therefore, this work aimed to characterize the phenolic composition and bioactive properties of cardoon blades throughout the phenological growth cycle. In addition to the structural variety of phytochemicals detected in the blade extracts, their antioxidant, anti-inflammatory, anti-proliferative, and antimicrobial properties were also highlighted. While immature material showed higher levels of phenolic compounds and greater potential to inhibit lipid peroxidation, samples at higher development stages had greater anti-proliferative, anti-inflammatory, and antimicrobial potential. These results demonstrate that the growth cycle influences the bioactive potential of cardoon blades and will be useful to establish suitable industrial applications, such as the development of ingredients for functional foods and nutraceuticals, among other products.

**Abstract:**

Cardoon (*Cynara cardunculus* var. *altilis*) blades were collected at sixteen sampling dates (B1–B16) to study the influence of the phenological growth stage on the phenolic composition and biological properties. Twenty phenolic compounds were identified, among which trans 3,4-*O*-dicaffeoylquinic acid, 5-*O*-caffeoylquinic acid, and luteolin-*O*-hexoside (39.6, 42.6, and 101.0 mg/g extract, respectively) were the main compounds. Immature blades (B3) had a higher content of phenolic compounds (178 mg/g extract) and a greater ability to inhibit the formation of thiobarbituric acid reactive substances (IC_50_ of 1.61 µg/mL). Samples at more advanced growth stages revealed a greater capacity to inhibit oxidative hemolysis (B8, IC_50_ of 25 and 47.4 µg/mL for Δ*t* of 60 and 120 min, respectively) and higher cytotoxic (B8–B13, GI_50_ between 7.1 and 17 µg/mL), anti-inflammatory (B13, IC_50_ of 10 µg/mL), and antibacterial activities. In turn, the antifungal activity varied depending on the tested fungi. All these results suggest that maturity influences the phenolic composition and bioactive properties of cardoon blades, which reveal great potential for the development of bioactive ingredients for food and pharmaceutical applications, among others.

## 1. Introduction

Cardoon (*Cynara cardunculus* L.) is a species characteristic of Mediterranean countries (Southern Europe and North Africa) currently found widespread all over the world. It belongs to the Asteraceae family, has a perennial growth habit, and comprises three varieties, namely *altilis*, commonly called cardoon or artichoke thistle, *sylvestris*, or wild cardoon, and *scolymus*, widely known as globe artichoke [1,2].

Cardoon is highly cultivated and consumed due to its health-promoting effects. It has been used in traditional medicine since ancient times for the treatment of liver illnesses and as an antidiabetic, anti-hemorrhoidal, choleretic, and cardiotonic agent [3,4]. At the industrial level, cardoon is applied in several sectors and is highly demanded due to its multifaceted potential. Characteristics such as its high yield, high resistance to adverse environmental conditions (e.g., high temperatures, stony and unproductive soils, and water shortage), low maintenance, as well as a wide variety of compounds with nutritional, pharmacological, and industrial interest have been crucial for the increasing interest that has been shown in this species [5,6].

It has been described in the literature that cardoon contains many important nutritional compounds, being a source of minerals, fibers, and inulin, as well as phenolic compounds, mainly flavonoids, caffeoylquinic and dicaffeoylquinic acid derivatives, and sesquiterpenes lactones [7,8,9]. This species also has several bioactive properties due to its wide variety of pharmacologically active compounds. Previous studies have attributed antioxidant, antibacterial, antiviral, anti-inflammatory, cytotoxic, and lipid-lowering properties to different cardoon vegetable tissues [10,11,12].

The fleshy stems and immature heads are the most consumed parts of cardoon, as they are used in many traditional recipes in Mediterranean cuisine, including in Algeria and Tunisia [3,4]. Its flowers are used as vegetable rennet in the production of protected designation of origin (PDO) cheeses [13]. Although the remaining parts of cardoon, namely leaf blades, stems, bracts, roots, and seeds are used in different industrial applications (e.g., biomass, biodiesel, paper pulp, oil for human consumption, and animal feed), the amount of by-products produced is still very high [3,14,15,16]. In particular, the blades and floral stems are usually discarded and therefore are considered waste [15,17]. The mentioned vegetable tissues can be a rich source of compounds of interest. Their exploitation is an asset for the valorization and complete characterization of the species and the simultaneous reduction of waste.

The tons of cardoon crop by-products have received particular attention due to the considerable growth in production and the need for alternative sources of nutrition due to the constant population increase. Some studies have already reported that plant by-products have a high amount of constituents with high bioactive potential, namely phenolic compounds with antioxidant and antimicrobial activity [3,14,15,18]. Simultaneously, parameters such as the genetic information, environmental conditions, geographical location, plant tissue, and harvest time influence the chemical composition and, consequently, the biological potential of the species [7,19,20].

Several studies have been carried out regarding the influence of the growth cycle of the different plant tissues of cardoon. Although the influence of the growth cycle has been proven both on the chemical composition and on the bioactive properties [8,19], the influence of the maturation cycle of the blades has not yet been described. Samples of heads, bracts, and petioles collected at the time of inflorescence (principal growth stage (PGS) between 5 and 6), show more promising bioactive properties, as well as higher levels of phenolic compounds [8,11]. Contrary to what was observed for seeds, in which the content of phenolic compounds increases with the growth cycle, samples with PGS 7/8 present greater bioactive potential [19]. To allow the reuse of by-products and the economic valorization of species within the context of the circular economy, it is important to study in-depth the biochemical composition of the species and the factors that can influence it, based on the observed differences.

The present study was carried out to evaluate the impact of harvest time on the phenolic composition and in vitro biological activities of cardoon blades and to identify alternative uses for this frequently discarded plant material. The plant material was collected from fully established plants throughout an entire growth cycle. The results are important for understanding the influence of the phenological growth stage on valuable bioactive constituents and promoting the valorization of this multifaceted crop.

## 2. Materials and Methods

### 2.1. Plant Material

Blades of *Cynara cardunculus* var. *altilis* DC cv. Bianco Avorio (Fratelli Ingepnoli Spa, Milano, Italy) were collected at the experimental field of the University of Thessaly in Velestino (22.756° E, 39.396° N), in Central Greece, during the growing period of 2017–2018. The blade samples were collected at sixteen harvesting dates. According to the Biologisch Bundesanstalt, Bundessortenamt, CHemische Industrie (BBCH) scale [21], the principal growth stages (PGS) of the samples were placed between 1 and 9. Samples B1, B2, and B3 were collected in September, October, and the beginning of November (all PGS 1), respectively. Sample B4 was collected at the end of November (PGS 2). Samples B5, B6, and B7 were collected at the beginning of January (PGS 3), February (PGS 3/4), and March (PGS 4), respectively. Samples B8 (PGS 4/5) and B9 (PGS 5) were collected at the beginning and at the end of April, respectively. Samples B10 (PGS 5/6) and B11 (PGS 6) were collected at the beginning and at the end of May. Sample B12 (PGS 6/7) was collected in June. Samples B13 (PGS 7/8) and B14 (PGS 8) were collected at the beginning and at the end of July, respectively. Samples B15 (PGS 8/9) and B16 (PGS 9) were collected at the beginning and at the end of August, respectively. The sampling dates and PGSs are presented in Table 1.

All blade samples were lyophilized (Sublimator model EKS, Christian Zirbus Co., Brunswick, Germany), reduced to a fine powder (~20 mesh) using a domestic grinder, and stored in an air-sealed bag under protection from light in a deep freezer (−80 °C) until analysis.

### 2.2. Extraction

Blades samples (1.5 g) were extracted under stirring (150 rpm) with 30 mL of EtOH/H_2_O (80:20, *v*/*v*) for 1 h at room temperature. The obtained solutions were filtered using Whatman paper No. 4 and the residues were re-extracted once under the same conditions. The combined extracts were concentrated at 40 °C under reduced pressure (Rotavapor Büchi R-210, Flawil, Switzerland) and the aqueous phase was frozen and lyophilized (FreeZone 4.5, Labconco, Kansas City, MO, USA) to perform the remaining analyses. The extraction yield was calculated and expressed as a percentage (%, *w*/*w*).

### 2.3. Polyphenolic Composition Analysis

The extracts were re-dissolved in EtOH:H_2_O (20:80, *v*/*v*) to obtain solutions at 10 mg/mL and filtered through 0.22 µm syringe filters. The phenolic compound’s composition was analyzed by high-performance liquid chromatography coupled to a diode array detector and electrospray ionization mass spectrometry (HPLC-DAD-ESI/MS) following the analytical conditions previously described by Bessada et al. [22]. Briefly, for the separation of the compounds, a Waters Spherisorb S3 ODS-2 C18 column (3 μm, 4.6 mm × 150 mm, Waters, Milford, MA, USA) was thermostatted at 35 °C. (A) 0.1% formic acid in water and (B) acetonitrile as solvents were used in the following isocratic gradient: 15% B (5 min), 15% B to 20% B (5 min), 20–25% B (10 min), 25–35% B (10 min), 35–50% B (10 min), and re-equilibration of the column, using a flow rate of 0.5 mL/min.

Tentative identification of phenolic compounds was accomplished through the comparison of the obtained chromatographic information (retention times and UV-Vis and mass spectra) with commercial standards and the available literature information. The quantification was achieved through the determination of the area of the peaks and their calibration curves prepared for the most similar commercial standards available (Extrasynthèse, Genay, France), e.g., compounds with peak number 1, 2, 3, 4, 5, 6, 7, 8, 9, 10, 11, 13, 14, 15, 16, 17, 19, and 20 were calibrated with chlorogenic acid (*y* = 168823*x* + 161172, *r*^2^ = 0.9999; limit of detection (LOD) = 0.20 µg/mL; limit of quantification (LOQ) = 0.68 µg/mL); compounds with peak number 12 and 18 were calibrated with quercetin-3-*O*-glucoside (*y* = 34843*x* − 160173, *r*^2^ = 0.9998; LOD = 0.21 µg/mL; LOQ = 0.71 µg/mL); in cases where the standard was not available, the quantification was performed using the most similar phenolic compound in terms of structural and similar chromatographic response. The results were presented as mg of equivalent of the most similar compound per g of extract.

### 2.4. In Vitro Evaluation of Biological Activities

#### 2.4.1. Antioxidant Activity

The antioxidant activity of the cardoon blade extracts was tested using two cell-based in vitro assays, which assess the ability to inhibit the formation of thiobarbituric acid reactive substances (TBARS) and the oxidative hemolysis (OxHLIA). The synthetic antioxidant Trolox (Fisher Scientific, Lisbon, Portugal) was used as a positive control.

The ability of the extracts to inhibit TBARS formation was evaluated following the procedure described in our previous work [19]. The extracts were re-dissolved in water to obtain a solution at 5 mg/mL that was further diluted to obtain the range of concentrations (1.2–625 µg/mL) to be tested. The results were expressed as the extract concentration that causes a 50% inhibition of the oxidative process (IC_50_ µg/mL).

The oxidative hemolysis assay allows the evaluation of the capacity of the extracts to inhibit the oxidative hemolysis of erythrocytes isolated from healthy sheep. The procedure was followed as described by [23]. The results were expressed as the extract concentration responsible for keeping 50% of the erythrocyte population intact (IC_50_ µg/mL) at Δ*t* of 60 and 120 min.

#### 2.4.2. Cytotoxic and Hepatotoxic Activities

Cardoon blades extracts were dissolved in water and diluted by successive dilutions to obtain the range of concentrations (0.125–8 mg/mL) to be tested. The different extract concentrations were incubated with the tested cell lines (190 µL, 10,000 cells/mL), being the range of final concentrations tested between 6.25 and 400 µg/mL. Four tumor cell lines were used: cervical carcinoma (HeLa), breast carcinoma (MCF-7), hepatocellular carcinoma (HepG2), and non-small cell lung cancer (NCI-H460). All the tested cell lines were commercially obtained from Leibniz-Institute DSMZ—German Collection of Microorganisms and Cell Cultures GmbH. The hepatotoxic potential was evaluated using a non-tumor porcine liver primary culture (PLP2); this culture was established by our team in the laboratory using porcine liver tissue, according to the procedure previously described [19]. The cytotoxic and hepatotoxic potentials were evaluated using the sulforhodamine B assay [24]. Ellipticine was used as a positive control (Sigma-Aldrich, St. Louis, MO, USA), and the cell suspension without any sample was used as a negative control. Results were expressed as the extract concentration responsible for 50% of cell growth inhibition (GI_50_, µg/mL).

#### 2.4.3. Anti-Inflammatory Activity

The anti-inflammatory activity was evaluated through the determination of the extract’s capacity to inhibit the lipopolysaccharide (LPS)-induced nitric oxide (NO) production by a murine macrophage cell line (RAW 264.7) and using the Griess Reagent System Kit (Promega, Madison, WI, USA) as previously described by Silva et al. [25]. Cardoon blade extracts were dissolved in water and further diluted to obtain the range of concentrations (0.125–8 mg/mL) to be tested. Dexamethasone (50 µM) (Sigma-Aldrich, Saint Louis, MO, USA) was used as a positive control, while cells without LPS were the negative control (Sigma-Aldrich, Saint Louis, MO, USA). Results were expressed as the extract concentration responsible for 50% of NO production inhibition (IC_50_, µg/mL).

#### 2.4.4. Antimicrobial Activity

The antimicrobial activity of the blade extracts was evaluated according to the procedure described by Fernandes et al. [26]. The extracts were re-dissolved in 5% dimethyl sulfoxide (DMSO) (Sigma-Aldrich, Saint Louis, MO, USA) and further diluted to obtain the range of concentrations to be tested. For evaluation of the antibacterial activity, the three Gram-positive (*Bacillus cereus* (food isolate), *Staphylococcus aureus* (ATCC 11632), and *Listeria monocytogenes* (NCTC 7973)) and three Gram-negative (*Escherichia coli* (ATCC 25922), *Enterobacter cloacae* (ATCC 35030), and *Salmonella* Typhimurium (ATCC 13311)) bacteria were selected. The antifungal activity was tested against the micromycetes *Aspergillus fumigatus* (human isolate), *Aspergillus versicolor* (ATCC 11730), *Aspergillus niger* (ATCC 6275), *Penicillium funiculosum* (ATCC 36839), *Penicillium ochrochloron* (ATCC 9112) and *Penicillium verrucosum* var. cyclopium (food isolate). The microorganisms were deposited at Mycological Laboratory, Department of Plant Physiology, Institute for Biological Research “Siniša Stanković”—National Institute of the Republic of Serbia, University of Belgrade, Serbia. As positive controls, the antibiotics ampicillin and streptomycin and the fungicides bifonazole and ketoconazole (acquired from Sigma-Aldrich, St. Louis, MO, USA) were used, while the negative control was 5% DMSO. The results were expressed as the minimal inhibitory (MIC), bactericidal (MBC), or fungicidal (MFC) concentrations (mg/mL).

### 2.5. Statistical Analysis

All experiments were performed in triplicate. The results were presented as the mean value ± standard deviation (except for antimicrobial activity). The obtained results were analyzed using SPSS Statistics (IBM SPSS Statistics for Mac OS, Version 26.0; IBM Corp., Armonk, NY, USA) by applying one-way analysis of variance (ANOVA) followed by Tukey’s HSD test, with α = 0.05.

## 3. Results and Discussion

### 3.1. Phenolic Compounds Composition

The phenolic profile of cardoon blades was characterized to evaluate the influence of the phenological growth stage. The chromatographic data (retention time, wavelength of maximum absorption, fragmentation pattern, and pseudomolecular ion) are presented in Table 2, and the content of each identified phenolic compound is shown in Table 3.

**Figure 1:**
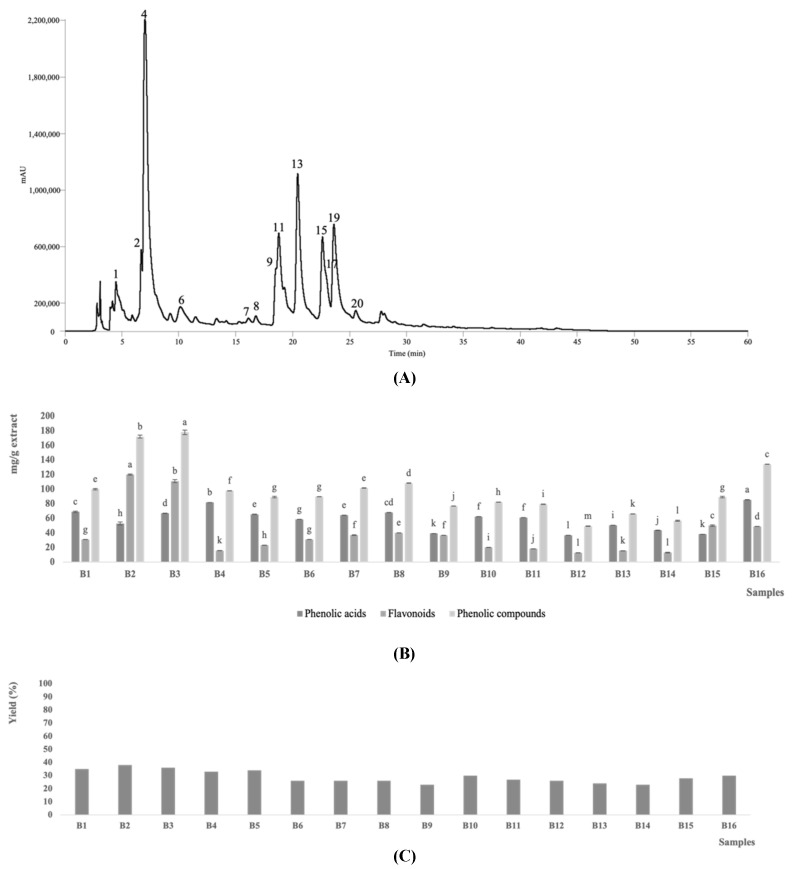
Representative phenolic profile of the *C. cardunculus* blade extracts (B6 sample) recorded at 280 nm. The phenolic compounds tentatively identified are described in Table 1 (**A**); total contents (mg/g extract) of phenolic acids, flavonoids, and phenolic compounds in the extracts of the sixteen *C. cardunculus* samples. For each group of compounds, different letters correspond to significant differences (*p* < 0.05) between samples (**B**). Yield of the tested blades’ extracts (**C**).

A representative chromatogram of the phenolic profile of cardoon blades is shown in Figure 1A, as is a graphical representation of the content (mg/g extract) in total phenolic acids, total flavonoids, and total phenolic compounds in all the hydroethanolic extracts studied in Figure 1B. Twenty phenolic compounds were tentatively identified, including 14 phenolic acid derivatives (peaks 1, 2, 3, 4, 5, 6, 8, 10, 13, 14, 15, 16, 17, and 20), and six flavonoid glycosides (peaks 7, 9, 11, 12, 18, and 19). Some of the compounds identified in the blade extracts were neither detected at early maturations states (e.g., samples B1–B3), nor at the senescence (e.g., samples B15 and B16), namely the compounds with the peak numbers 1, 2, 4, 6, 7, 8, 9, 11, 13, 15, 17, 19, 20. Of the twenty identified phenolic compounds, most of them were phenolic acid derivatives, with the 5-*O*-caffeoylquinic acid (13.3–42.6 mg/g extract), luteolin-*O*-hexoside (24.2–101 mg/g extract), *trans* 1,5-di-*O*-caffeoylquinic acid (18.17–39.6 mg/g extract) and luteolin-*O*-malonyl hexoside derivative I (6.7–54.0 mg/g extract) being the compounds with the highest overall abundance. Although several authors have already studied the phenolic composition of different cardoon tissues (namely heads, bracts, inflorescences, seeds, and petioles), the influence that the maturation stage has on the phenolic composition of blades has never been previously described.

The phenolic composition of cardoon blades was very similar to the composition of petioles, previously described by Mandim et al. [8]. The peak numbers 2, 3, 5, 7, 8, 9, 11, 18, 19, and 20 (namely protocatechuic acid, *cis* 4-*O*-caffeoylquinic acid, *trans* 5-*O*-caffeoylquinic acid, eriodictyol-*O*-hexuronoside, *cis* 1,3-di-*O*-caffeoylquinic acid, luteolin-*O*-hexuronoside derivative I and derivative II, luteolin-*O*-malonyl hexoside derivative I and derivative II, and *trans* 3,5-di-*O*-caffeoylquinic acid, respectively) were previously identified in cardoon petioles [8]. Peak number 1 (3-*O*-caffeoylquinic acid) was previously identified in cardoon tissues [8,15,27], while peak number 4 (*cis* 5-*O*-caffeoylquinic acid) was previously detected in cardoon heads, seeds, and bracts [8,9,12,19]. The compound identified with peak number 6 (caffeoylquinic acid derivative) was also previously identified [15,28]. Peaks number 12 and 16 (luteolin-*O*-hexoside and *trans* 3,4-di-*O*-caffeoylquinic acid) were reported in cardoon bracts and heads and seeds, respectively [8,11,19]. Similarly, peaks number 10, 14, and 17 (*trans* 1,3-Di-*O*-caffeoylquinic acid, *trans* 1,5-di-*O*-caffeoylquinic acid, and *cis* 3,5-*O*-diccafeoylquinic acid, respectively) were also recorded in cardoon different parts [8,27,28]. Regarding the peaks identified with the numbers 13 and 15 (*cis* and *trans* 1,5-di-*O*-caffeoylquinic acids, respectively) both of them were previously detected in cardoon [8,17].

Early maturation stages (e.g., samples B2 and B3 corresponding to PGS 1) are those with the highest content of total phenolic compounds (172 and 178 mg/g extract, respectively, Figure 1B). As observed in blades, tissues of heads and bracts at younger maturation states also presented the highest content of phenolic compounds [11,12]. On the other hand, seed samples have higher amounts of phenolic compounds at more advanced growth stages [19]. The influence of the maturation stage on the polyphenolic composition of blades was proven in this work, but other parameters such as genetic information, tissues viability, and different parts of the plant, may also have an influence on this parameter [7,28,29]. The characterization and evaluation of the influence of all the parameters mentioned are extremely important for the proper use and valorization of all the constituents of the species.

### 3.2. Bioactive Properties

#### 3.2.1. Antioxidant Activity

The results regarding the antioxidant activity of cardoon blades are presented in Table 4. All the analyzed samples revealed capacity to inhibit the oxidative process in both assays. The maturity stage of cardoon blades revealed a statistically significant (*p*-value < 0.05) influence on the antioxidant potential of the tested samples. For the TBARS assay, lower IC_50_ values (highest antioxidant activity) were observed for the samples collected at early maturity. In particular, sample B3 revealed the lowest IC_50_ value (1.61 µg/mL), followed by samples B2 (IC_50_ = 3.0 µg/mL) and B1 (IC_50_ = 5.2 µg/mL). Better results for the TBARS assay were obtained for the samples with higher content in phenolic compounds, a finding that suggests a positive correlation between these two parameters. Moreover, the high content of luteolin hexoside and luteolin-O-malonyl hexoside derivative I in both samples indicates the possible involvement of these phenolic compounds in the TBARS formation inhibition of cardoon petioles. On the other hand, sample B12 (PGS 6/7) revealed the highest IC_50_ value (198 µg/mL) and therefore, the lowest capacity to inhibit lipid peroxidation. Moreover, several cardoon blade samples had a higher capacity to inhibit lipid oxidation than the antioxidant Trolox used as a positive control, namely samples B1–B3, B15, and B16, since they demonstrated lower IC_50_ values.

For OxHLIA, blades collected at intermedium maturity were the ones that revealed the highest capacity to inhibit oxidative hemolysis, namely samples B4 (IC_50_ = 26 and 45 µg/mL for Δ*t* of 60 and 120 min, respectively) and B8 (IC_50_ = 25 and 47.4 µg/mL for Δ*t* of 60 and 120 min, respectively). This finding suggested the possible correlation of 5-*O*-caffeoylquinic and *cis* 3,5-*O*-dicaffeoylquinic acids with antihemolytic activity. In contrast, the highest IC_50_ values were obtained for the blades collected at late maturity and PGS 9 (sample B16) (IC_50_ = 112 and 183 µg/mL for Δ*t* of 60 and 120 min, respectively). Although some samples had an IC_50_ similar to Trolox (namely samples B4 and B8), none of them had a superior potential to protect the erythrocyte membranes from hemolysis induced by the free radical generator 2,2′-azobis(2-amidinopropane) dihydrochloride (AAPH).

The antioxidant activity of the different plant tissues of cardoon has been studied, mainly through the 2,2-diphenyl-1-picrylhydrazyl (DPPH) assay. Although the influence of the maturation stage in cardoon seeds, heads, petioles, and bracts has already been proven, to the best of the authors’ knowledge, the influence of the blade’s maturity on this bioactive property has never been previously described. According to the literature, findings similar to those observed in our study for blades were also recorded for immature bracts and petioles (PGS 1), which revealed greater efficiency in inhibiting lipid peroxidation [8,11]. On the contrary, the activity of seeds was higher when harvested at a more advanced maturation stage [19]. Moreover, bracts and heads harvested at intermediate growth stages (PGS 5 and 6/7, respectively) revealed better bioactivities [11,29].

For OxHLIA, the results were not so homogeneous, with the petioles at early (sample B4; PGS 2) and intermediate (sample B8; PGS 4/5) maturation stages showing better activities [8], as herein verified for blades. On the contrary, for heads (PGS 5) [12], bracts (PGS 8) [11], and seeds (PGS 7/8) [19], higher activities were observed for samples with a higher degree of maturation. In addition to the maturation stage, characteristics such as genetic information, geographic location, and plant tissue, also influence the bioactive potential of cardoon [7,12]. According to Pandino et al. [17], who studied the antioxidant potential of ten cardoon genotypes and tissues, the floral stem of cultivated cardoon presents greater activity compared to wild samples, as well as higher antioxidant potential than the leaf. However, according to the literature, there are contradictory results regarding the antioxidant potential of phenolic compounds, which is in agreement with our results, since different classes of compounds were demonstrated to be responsible for the antioxidant activity observed in the different assays used [26,29]. This finding also justifies the use of targeted compounds for specific bioactive compounds, which helps to valorize natural matrices, especially discarded material as in the case of our study [30].

Moreover, the present study enabled us to prove the influence of the maturity stage, highlighting younger plant tissues as those that should be explored in the search for molecules with antioxidant power.

#### 3.2.2. Cytotoxic Activity

The data regarding the cytotoxic activity of cardoon blades are presented in Table 5. The results were presented as the extract concentration responsible for the inhibition of cell proliferation by 50% (GI_50_). Although the activities were different among the cell lines tested, the intermediate maturation stages (samples B8–B13 corresponding to PGSs between 4/5 and 7/8) were those that presented a greater cytotoxic potential with the lowest GI_50_ values (between 7.1 and 17 µg/mL) against most of the tested cell lines. In contrast, samples B7 (PGS 4) and B16 (PGS 9) showed the highest GI_50_ values (between 38 and 95 µg/mL) and, therefore, a lower cytotoxic activity. Among the tumor cell lines tested, the one that revealed high susceptibility to the cardoon blades was the hepatocellular carcinoma cell line (HepG2), as it revealed the lowest GI_50_ values.

It is interesting to note that among the samples with the highest overall activity was sample B12, which contained the lowest amounts of total phenolic compounds. This finding indicates that despite the significance of phenolic compounds for the bioactive profile of petioles, other compounds not determined in our study should be also considered responsible for cytotoxic effects. This contradiction is not unusual in the literature, where variable results regarding the phenolic compounds content and its importance in bioactivities have been reported, since several factors may affect the chemical composition of plant tissues and thus their bioactive potential [28,30].

The influence of harvest time on the cytotoxic potential of cardoon blades has never been previously described. Previous studies with different growth stages of seeds, capitulum, bracts, and petioles proved the influence of the maturation stage on the cytotoxic potential of cardoon. In agreement with the results obtained in this study, petioles in intermediate maturation state (PGS between 5 and 6/7), also stood out with lower GI_50_ values [8]. On the other hand, seeds in more advanced stages of maturation (PGS 8) [19], and heads and bracts in early stages of maturation (PGS 5) were those with greater activity [11,12]. Moreover, lipophilic extracts obtained from leaves and florets revealed cytotoxic potential against triple-negative breast cancer (TNBC), with leaf extracts exhibiting a higher capacity to inhibit cellular viability than florets [31].

#### 3.2.3. Hepatotoxic Activity

The obtained results for the evaluation of the hepatotoxic activity of hydroethanolic extracts of cardoon blades against the PLP2 cell line are presented in Table 5. Although the studied extracts revealed activity for this cell line, the GI_50_ values obtained are higher than those obtained for the tumor cell lines tested, except for the samples B3, B7, and B16. In general, the studied extracts have greater cytotoxic activity against tumor cell lines. This fact is important since it demonstrates that the cardoon blades can be a source of biomolecules with anticancer potential, without revealing toxicity to non-tumor liver cells. The absence of toxicity to liver cells has been previously described in extracts of cardoon obtained from seeds [19,29], immature bracts [11] petioles [8], and heads at intermediate maturation stages [12], which further justify the safety of the various plant parts of the species.

#### 3.2.4. Anti-Inflammatory Activity

The anti-inflammatory activity was determined through the ability of cardoon blade extracts to inhibit the formation of the pro-inflammatory mediator NO by the LPS-stimulated murine macrophage cell line (RAW 246.7) and the results are presented in Table 6. All studied maturation stages revealed anti-inflammatory potential. In particular, samples B11, B12, and B13 were those that demonstrated a better ability to inhibit NO production, with IC_50_ values (IC_50_ = 13.5, 12, and 10 µg/mL, respectively) lower than the positive control used (dexamethasone, IC_50_ = 16 µg/mL). The fact that these samples contained mostly 5-*O*-Caffeoylquinic acid and *cis* 3,5-O-dicaffeoylquinic acid, which are common in most of the petiole samples, indicates that probably other compounds not detected in our study or phenolic compounds present in low amounts are significant for the observed anti-inflammatory activity. On the other hand, sample B7 (PGS 3) showed the least promising results, with a higher IC_50_ value (IC_50_ = 72 µg/mL), when compared to the other samples studied.

The studies regarding the anti-inflammatory potential of different plant tissues of cardoon are very scarce [12,29]. Previous reports that evaluated the influence of the maturation state of cardoon tissues on the anti-inflammatory potential found that petioles [8], bracts [11], and heads [12] with PGS 5 had the most interesting anti-inflammatory potential among the samples analyzed. This fact agrees with the results obtained in this study and further suggests that the aforementioned vegetable tissues are those that present high anti-inflammatory potential, and therefore are of great interest in the exploration of bioactive compounds for this purpose. On the contrary, petioles at PGS 3 [19] demonstrated lower anti-inflammatory potential, contrary to what was observed for bracts and heads [11,12], where samples with PGS between 5/6 and 8 showed the lowest anti-inflammatory potential, and therefore they were those samples of least interest for the exploration of compounds with anti-inflammatory power. The anti-inflammatory potential of cardoon was also evaluated in hydroethanolic extracts of various *Scolymus* species through an in vivo assay of the CARR test and it was suggested that the anti-inflammatory potential and the amount of phenolic compounds are directly correlated [32]. In another study where the influence of the viability of cardoon seeds was studied, no anti-inflammatory potential was observed in both studied samples [29]. To the best of the authors’ knowledge, the study of the influence of blades’ maturation stage throughout their entire growth cycle on their anti-inflammatory potential has never been previously described. Therefore, the presented results could be useful for the valorization of the species through the isolation of targeted bioactive compounds.

#### 3.2.5. Antimicrobial Activity

The antibacterial and antifungal activity of cardoon blades were evaluated using several bacteria and fungi strains and the obtained results are presented in Table 7 and Table 8, respectively. The results were expressed as the minimal inhibitory (MIC), bactericidal (MBC), and fungicidal (MFC) concentrations. All the studied extracts had the capacity to inhibit bacterial growth and cause the death of the studied bacterial strains. The MIC values obtained for Gram-positive bacteria were lower than those obtained for Gram negative ones. This fact is in agreement with previous studies, in which Gram-positive bacteria were more susceptible than the Gram-negative ones. The bacteria strain that revealed the highest susceptibility to cardoon bracts extracts was *A. fumigatus*, which recorded the lowest MIC values (between 0.43 and 3.57 mg/mL). Although the antibacterial potential is influenced by the bacteria strain under analysis, the extract B13 (PGS 7/8) was the one with the most promising activity, since it showed the lowest MIC values for four of the six bacteria studied (i.e., *S. aureus*, *E. cloacae*, *E. coli*, and *S. Typhimurium*). Other samples that showed promising results were samples B3 and B12, which were effective against *L. monocytogenes* and *B. cereus* (lowest MIC values), respectively. The mentioned samples (B3, B12, B13) varied in terms of phenolic compounds composition which indicates either a variable effect of specific compounds against specific bacteria or the presence of other bioactive compounds not detected in our study. However, in order to make safe conclusions regarding the antibacterial effect of specific compounds, the isolated pure compound has to be tested.

None of the tested samples revealed lower MIC values and therefore higher antibacterial capacity than the positive control used (commercial antibiotics, i.e., streptomycin and ampicillin). The obtained results agree with the findings described in [19], where the authors studied the influence of the maturation stage of cardoon seeds and the highest antibacterial activity was also obtained for seeds collected at PGS 7/8 (MIC values between 0.80 and 1.60 mg/mL). Compared to other plant tissues of cardoon, the MIC values obtained are lower than those shown by seeds [19], petioles [8], bracts [11], heads [12], as well as viable and non-viable seeds [29]. The results obtained in this work further suggest the influence that parameters such as maturation stage, plant tissue, genetic background, and tissue viability may have on the antibacterial potential of this species.

The antifungal activity was also evaluated against six fungi strains. The obtained MIC and MFC values are presented in Table 8. The antifungal potential differed between the tested samples, without any growth stage standing out from the others studied, while a fungus dependent activity was recorded. In particular, samples B14 and B12 showed the greatest potential against *A. fumigatus* and *A. niger*, respectively, while sample B11 was the most effective against *A. versicolor* and *P. funiculosum* and sample B4 against *P. ochrochloron* and *P. verrucosum* var. *cyclopium*. The fact that all these samples have in common a high content of 5-*O*-caffeoylquinic acid indicates the importance of this specific compound in the bioactive profile of the species. Moreover, the antifungal activity of extracts from different parts of cardoon has been previously confirmed and the influence of parameters such as maturation state, genetic information, and plant tissues used on this potential has been verified [19,28].

## 4. Conclusions

The maturation stage of cardoon blades influences the phenolic profile and in vitro bioactive properties. Samples in earlier maturation stages (sample B3) had the highest contents of phenolic compounds, namely luteolin-*O*-hexoside and *trans* 3,4-*O*-dicaffeoylquinic acid, which were detected in larger amounts. Additionally, sample B3 (PGS 1) showed the greatest potential to inhibit lipid peroxidation by the TBARS assay. In contrast, samples at intermediate to late stages of maturation presented the greatest potential to inhibit oxidative hemolysis (samples B4 and B8), tumor cell growth (samples B8–13), and production of the inflammatory mediator NO (samples B11–14). Regarding antibacterial and antifungal activities, the more mature samples had higher efficiency in inhibiting the growth of the tested bacterial strain than the petioles collected earlier. On the other hand, antifungal activity varied depending on the tested fungi. Overall, this study contributes to a more complete and in-depth knowledge of the multifaceted potential associated with cardoon, in particular its blades, which constitute a significant portion of discarded biomass. Therefore, it can be suggested that this by-product can be upcycled for medicinal and nutraceutical purposes due to its demonstrated bioactive potential. Additionally, the valorization of cardoon blades can be promoted through the circular economy and sustainable production concepts.

## Figures and Tables

**Table 1:** Sampling dates and the corresponding principal growth stages (PGS) throughout the growth cycle.

Sampling Date	Principal Growth Stage
10 September	PGS 1
10 October	
10 November	
30 November	PGS 2
9 January	PGS 3
8 February	PGS 3/4
8 March	PGS 4
7 April	PGS 4/5
26 April	PGS 5
10 May	PGS 5/6
24 May	PGS 6
12 June	PGS 6/7
4 July	PGS 7/8
18 July	
9 August	PGS 8
29 August	PGS 9

**Table 2:** Phenolic compounds tentatively identified in the hydroethanolic extracts of *Cynara cardunculus* L. var *altilis* blades collected at different growth stages. The retention time (Rt), wavelength of maximum absorption (λ_max_) in the UV-Vis region, and mass spectral data are presented.

Peak	Rt (min)	λ_max_ (nm)	[M-H]^−^ (*m*/*z*)	MS^2^ (*m*/*z*)	Tentative Identification
1	4.50	327	353	191 (100), 179 (5), 161 (5), 135 (5)	3-*O*-Caffeoylquinic acid
2	6.69	266	153	109(100)	Protocatechuic acid
3	6.80	326	353	173 (100), 179 (11), 191 (10), 161 (5), 135 (5)	4-*O*-Caffeoylquinic acid
4	7.01	326	353	191 (100), 179 (10), 161 (5), 135 (5)	*cis* 5-*O*-Caffeoylquinic acid
5	7.11	326	353	191 (100), 179 (7), 173 (5), 135 (5)	*trans* 5-*O*-Caffeoylquinic acid
6	10.43	326	353	191 (100), 179 (11), 161 (5), 135 (5)	Caffeoylquinic acid derivate
7	16.13	285/sh324	463	287 (100)	Eriodictyol-*O*-hexuronoside
8	16.83	322	515	353 (100), 335 (26), 191 (64), 17 9(10)	*cis* 1,3-Di-*O*-caffeoylquinic acid
9	18.49	345	461	285 (100)	Luteolin-*O*-hexuronoside derivative I
10	18.70	334	515	353 (100), 179 (35), 173 (29), 353 (10), 191 (10), 135 (8), 161 (5)	*trans* 1,3-Di-*O*-caffeyolquinic acid
11	18.78	345	461	285 (100)	Luteolin-*O*-hexuronoside derivative II
12	18.93	340	447	285 (100)	Luteolin-*O*-hexoside
13	20.41	344	515	353 (100), 191 (12), 335 (10)	*cis* 1,5-Di-*O*-cafffeoylquinic acid
14	20.50	328	515	353 (100), 191 (5), 335 (12)	*trans* 1,5-Di-*O*-cafffeoylquinic acid
15	22.66	329	515	353 (100), 335 (5), 229 (3), 255 (5), 203 (6), 191 (69), 179 (12), 173 (4, MS^3^ base peak)	*cis* 3,4-Di-*O*-cafffeoylquinic acid
16	22.70	329	515	353 (100), 335 (5), 229 (3), 255 (6), 203 (3), 191 (76), 179 (11), 173 (5, MS^3^ base peak)	*trans* 3,4-Di-*O*-cafffeoylquinic acid
17	22.89	329	515	353 (100), 335 (32), 191 (20), 179 (12)	*cis* 3,5-Di-*O*-cafffeoylquinic acid
18	23.65	332	533	489 (100), 285 (20)	Luteolin-*O*-malonyl hexoside derivative I
19	23.67	346	533	489 (50), 447 (5), 285 (100)	Luteolin-*O*-malonyl hexoside derivative II
20	25.60	330	515	353 (100), 191 (13, MS^3^ base peak)	*trans* 3,5-Di-*O*-caffeolyquinic acid

**Table 3:** Content (mg/g extract) of the phenolic compounds identified in the hydroethanolic extracts of *Cynara cardunculus* L. var. *altilis* blades collected at different growth stages.

Sample	Compound 1	Compound 2	Compound 3	Compound 4	Compound 5	Compound 6	Compound 7	Compound 8	Compound 9	Compound 10
B1	n.d.	n.d.	3.5 ± 0.1 ^d^	n.d.	25.5 ± 0.6 ^a^	n.d.	n.d.	n.d.	n.d.	3.08 ± 0.05 ^d^
B2	n.d.	n.d.	3.81 ± 0.02 ^c^	n.d.	3.33 ± 0.03 ^e^	n.d.	n.d.	n.d.	n.d.	3.4 ± 0.1 ^c^
B3	n.d.	n.d.	5.4 ± 0.2 ^b^	n.d.	19.5 ± 0.3 ^c^	n.d.	n.d.	n.d.	n.d.	3.6 ± 0.1 ^b^
B4	1.530 ± 0.002 ^e^	1.68 ± 0.04 ^b^	n.d.	42.6 ± 0.1 ^a^	n.d.	2.6 ± 0.1 ^c^	0.174 ± 0.001 ^h^	1.241 ± 0.003 ^c^	2.988 ± 0.003 ^d^	n.d.
B5	1.212 ± 0.005 ^f^	1.383 ± 0.002 ^d^	n.d.	36.63 ± 0.02 ^b^	n.d.	2.124 ± 0.005 ^ef^	0.8220 ± 0.0003 ^e^	0.66 ± 0.02 ^j^	2.7 ± 0.1 ^e^	n.d.
B6	2.90 ± 0.01 ^d^	2.53 ± 0.03 ^a^	n.d.	35.47 ± 0.02 ^c^	n.d.	4.06 ± 0.02 ^a^	0.83 ± 0.01 ^e^	0.86 ± 0.01 ^g^	0.417 ± 0.005 ^k^	n.d.
B7	0.584 ± 0.003 ^i^	1.18 ± 0.02 ^e^	n.d.	31.05 ± 0.05 ^d^	n.d.	3.120 ± 0.003 ^b^	0.934 ± 0.001 ^c^	1.04 ± 0.01 ^e^	2.23 ± 0.02 ^f^	n.d.
B8	1.206 ± 0.001 ^f^	0.825 ± 0.003 ^h^	n.d.	26.1 ± 0.2 ^f^	n.d.	2.01 ± 0.04 ^g^	0.979 ± 0.002 ^b^	0.97 ± 0.01 ^f^	3.19 ± 0.02 ^c^	n.d.
B9	0.88 ± 0.01 ^h^	1.53 ± 0.04 ^c^	n.d.	21.21 ± 0.04 ^h^	n.d.	2.171 ± 0.004 ^e^	1.05 ± 0.01 ^a^	1.05 ± 0.01 ^e^	7.3 ± 0.1 ^a^	n.d.
B10	3.15 ± 0.03 ^c^	1.38 ± 0.01 ^d^	n.d.	29.8 ± 0.3 ^e^	n.d.	2.52 ± 0.05 ^d^	0.86 ± 0.01 ^d^	0.707 ± 0.005 ^i^	1.946 ± 0.001 ^h^	n.d.
B11	5.56 ± 0.01 ^b^	1.15 ± 0.02 ^e^	n.d.	24.6 ± 0.1 ^g^	n.d.	1.33 ± 0.02 ^h^	0.636 ± 0.002 ^g^	0.761 ± 0.003 ^h^	1.45 ± 0.01 ^j^	n.d.
B12	6.6 ± 0.1 ^a^	1.061 ± 0.003 ^f^	n.d.	20.5 ± 0.3 ^j^	n.d.	1.31 ± 0.01 ^h^	0.82 ± 0.02 ^e^	1.193 ± 0.003 ^d^	1.68 ± 0.03 ^i^	n.d.
B13	1.1623 ± 0.0003 ^g^	1.416 ± 0.003 ^d^	n.d.	20.83 ± 0.03 ^i^	n.d.	1.98 ± 0.01 ^g^	0.93 ± 0.01 ^c^	1.37 ± 0.02 ^a^	3.93 ± 0.01 ^b^	n.d.
B14	0.91 ± 0.02 ^h^	0.89 ± 0.01 ^g^	n.d.	13.3 ± 0.3 ^k^	n.d.	2.09 ± 0.03 ^f^	0.78 ± 0.01 ^f^	1.28 ± 0.01 ^b^	2.1615 ± 0.0004 ^g^	n.d.
B15	n.d.	n.d.	3.6 ± 0.1 ^d^	n.d.	11.63 ± 0.02 ^d^	n.d.	n.d.	n.d.	n.d.	1.9 ± 0.1 ^e^
B16	n.d.	n.d.	8.2 ± 0.1 ^a^	n.d.	23.3 ± 0.03 ^b^	n.d.	n.d.	n.d.	n.d.	9.03 ± 0.02 ^a^
	**Compound 11**	**Compound 12**	**Compound 13**	**Compound 14**	**Compound 15**	**Compound 16**	**Compound 17**	**Compound 18**	**Compound 19**	**Compound 20**
B1	n.d.	24.2 ± 0.1 ^e^	n.d.	33.5 ± 0.5 ^c^	n.d.	3.82 ± 0.01 ^d^	n.d.	6.70 ± 0.01 ^e^	n.d.	n.d.
B2	n.d.	101 ± 1 ^a^	n.d.	36 ± 2 ^b^	n.d.	6.0 ± 0.3 ^b^	n.d.	18.6 ± 0.3 ^b^	n.d.	n.d.
B3	n.d.	56.5 ± 2.5 ^b^	n.d.	31.1 ± 0.2 ^d^	n.d.	7.4 ± 0.2 ^a^	n.d.	54 ± 1 ^a^	n.d.	n.d.
B4	3.335 ± 0.003 ^j^	n.d.	25.9 ± 0.1 ^b^	n.d.	3.8 ± 0.1 ^f^	n.d.	1.70 ± 0.01 ^g^	n.d.	9.4 ± 0.1 ^c^	0.80 ± 0.02 ^e^
B5	10.6 ± 0.1 ^f^	n.d.	18.4 ± 0.5 ^e^	n.d.	2.6 ± 0.1 ^g^	n.d.	1.60 ± 0.01 ^h^	n.d.	9.07 ± 0.02 ^d^	0.84 ± 0.01 ^d^
B6	13.9 ± 0.1 ^d^	n.d.	1.982 ± 0.001 ^h^	n.d.	7.34 ± 0.03 ^a^	n.d.	2.174 ± 0.002 ^b^	n.d.	15.9 ± 0. 2 ^b^	1.442 ± 0.003 ^a^
B7	18.0 ± 0.3 ^b^	n.d.	19.0 ± 0.2 ^d^	n.d.	5.27 ± 0.01 ^b^	n.d.	1.67 ± 0.02 ^g^	n.d.	16.0 ± 0.1 ^b^	1.43 ± 0.03 ^a^
B8	16.36 ± 0.04 ^c^	n.d.	31.73 ± 0.04 ^a^	n.d.	2.502 ± 0.002 ^h^	n.d.	1.49 ± 0.01 ^i^	n.d.	19.6 ± 0.1 ^a^	1.29 ± 0.01 ^b^
B9	20.06 ± 0.03 ^a^	n.d.	7.14 ± 0.04 ^g^	n.d.	2.38 ± 0.02 ^i^	n.d.	1.99 ± 0.01 ^d^	n.d.	9.27 ± 0.03 ^c^	0.90 ± 0.01 ^c^
B10	12.1 ± 0.1 ^e^	n.d.	17.291 ± 0.002 ^f^	n.d.	3.98 ± 0.002 ^e^	n.d.	2.0 ± 0.1 ^c^	n.d.	5.17 ± 0.02 ^f^	1.29 ± 0.02 ^b^
B11	10.686 ± 0.003 ^f^	n.d.	20.8 ± 0.1 ^c^	n.d.	4.13 ± 0.02 ^d^	n.d.	1.83 ± 0.04 ^f^	n.d.	5.5 ± 0.1 ^e^	0.84 ± 0.02 ^d^
B12	8.3 ± 0.1 ^h^	n.d.	1.697 ± 0.001 ^h^	n.d.	1.81 ± 0.04 ^k^	n.d.	1.88 ± 0.04 ^e^	n.d.	1.89 ± 0.01 ^h^	0.63 ± 0.01 ^h^
B13	9.09 ± 0.04 ^g^	n.d.	19.12 ± 0.01 ^d^	n.d.	1.974 ± 0.002 ^j^	n.d.	2.020 ± 0.002 ^cd^	n.d.	1.640 ± 0.003 ^i^	0.719 ± 0.002 ^g^
B14	7.8 ± 0.3 ^i^	n.d.	17.5 ± 0.1 ^f^	n.d.	4.66 ± 0.01 ^c^	n.d.	2.28 ± 0.03 ^a^	n.d.	2.2 ± 0.1 ^g^	0.76 ± 0.02 ^f^
B15	n.d.	41 ± 1 ^c^	n.d.	18.17 ± 0.05 ^e^	n.d.	2.95 ± 0.03 ^e^	n.d.	9.1 ± 0.5 ^d^	n.d.	n.d.
B16	n.d.	39.7 ± 0.4 ^d^	n.d.	39.6 ± 0.5 ^a^	n.d.	5.23 ± 0.01 ^c^	n.d.	9.1 ± 0.3 ^d^	n.d.	n.d.

Results are expressed as mean ± standard deviation. n.d.: not detected. In each column, different letters correspond to significant differences (*p* < 0.05) between samples.

**Table 4:** Antioxidant activity of the hydroethanolic extracts of *Cynara cardunculus* L. var *altilis* blades collected at different growth stages.

Sample	TBARS (IC_50_, µg/mL)	OxHLIA (IC_50_, µg/mL)	
		Δ*t* 60 min	Δ*t* 120 min
B1	5.2 ± 0.1 ^l^	92 ± 2 ^b^	126 ± 3 ^bc^
B2	3.0 ± 0.1 ^m^	96 ± 1 ^b^	137 ± 2 ^b^
B3	1.61 ± 0.03 ^m^	99 ± 7 ^b^	173 ± 8 ^a^
B4	46.7 ± 0.2 ^g^	26 ± 1 ^hi^	45 ± 1 ^i^
B5	54 ± 2 ^e^	52 ± 1 ^defg^	95 ± 2 ^fg^
B6	49.4 ± 0.3 ^f^	59 ± 2 ^cd^	111 ± 1 ^de^
B7	43.0 ± 0.3 ^h^	44 ± 1 ^g^	78 ± 1 ^h^
B8	11.6 ± 0.1 ^j^	25 ± 1 ^i^	47.4 ± 0.5 ^i^
B9	85 ± 1 ^c^	58 ± 2 ^cde^	101 ± 3 ^efg^
B10	38.4 ± 0.1 ^ij^	33 ± 1 ^h^	58 ± 1 ^i^
B11	81.4 ± 0.1 ^d^	60 ± 3 ^cd^	116 ± 6 ^cd^
B12	198 ± 1 ^a^	49.4 ± 0.3 ^fg^	88 ± 6 ^gh^
B13	81.9 ± 0.2 ^d^	50 ± 3 ^efg^	95 ± 7 ^fg^
B14	126 ± 3 ^b^	54 ± 2 ^cdef^	103 ± 3 ^def^
B15	6.01 ± 0.04 ^l^	62 ± 2 ^c^	114 ± 2 ^cde^
B16	8.7 ± 0.2 ^k^	112 ± 2 ^a^	183 ± 6 ^a^
Trolox	9.1 ± 0.3	21.2 ± 0.7	41.1 ± 0.8

Results are expressed as mean ± standard deviation. In each column, different letters correspond to significant differences (*p* < 0.05) between samples. IC_50_ values correspond to the extract concentration needed to inhibit by 50% the formation of thiobarbituric acid reactive substances (TBARS) and oxidative hemolysis (OxHLIA).

**Table 5:** Cytotoxic activity of the hydroethanolic extracts of *Cynara cardunculus* L. var *altilis* blades collected at different growth stages.

Sample	Cytotoxic Activity (GI_50_, µg/mL)				
	MCF-7	NCI-H460	HeLa	HepG2	PLP2
B1	30 ± 1 ^d^	27 ± 2 ^g^	24 ± 1 ^c^	21 ± 2 ^de^	61 ± 2 ^c^
B2	24 ± 1 ^e^	20.5 ± 0.9 ^h^	23 ± 1 ^cd^	25 ± 2 ^c^	41 ± 1 ^e^
B3	58 ± 4 ^b^	53 ± 2 ^c^	44.2 ± 0.4 ^b^	36 ± 1 ^b^	80 ± 3 ^b^
B4	38 ± 3 ^c^	47 ± 1 ^d^	20 ± 2 ^de^	23 ± 1 ^cd^	51 ± 1 ^d^
B5	42 ± 3 ^c^	40.7 ± 1.5 ^e^	16 ± 1 ^g^	45 ± 4 ^a^	52 ± 1 ^d^
B6	24.5 ± 1.2 ^e^	33.5 ± 1.0 ^f^	20 ± 1 ^ef^	15 ± 1 ^ef^	41 ± 4 ^e^
B7	87 ± 4 ^a^	89 ± 8 ^a^	9.3 ± 0.5 ^h^	18 ± 2 ^e^	95 ± 2 ^a^
B8	8 ± 1 ^h^	8.7 ± 0.4 ^j^	10.0 ± 0.3 ^h^	13 ± 1 ^fg^	17.9 ± 1.5 ^h^
B9	10.1 ± 0.3 ^h^	40 ± 1 ^e^	9 ± 1 ^h^	11.1 ± 0.4 ^gh^	42 ± 2 ^e^
B10	16 ± 1 ^g^	16 ± 1 ^hi^	10 ± 1 ^h^	16 ± 1 ^ef^	24.1 ± 2.5 ^g^
B11	7.1 ± 0.5 ^h^	15.0 ± 0.4 ^i^	11 ± 1 ^h^	13 ± 1 ^fg^	17.1 ± 1.5 ^h^
B12	10 ± 1 ^h^	10 ± 1 ^j^	18 ± 2 ^fg^	9.1 ± 0.4 ^h^	21 ± 2 ^gh^
B13	9.0 ± 0.8 ^h^	12 ± 1 ^ij^	9.0 ± 0.4 ^h^	9 ± 1 ^h^	17 ± 2 ^h^
B14	19 ± 1 ^fg^	15 ± 1 ^i^	16 ± 1 ^g^	16 ± 1 ^ef^	30.5 ± 1.1 ^f^
B15	22 ± 1 ^ef^	26 ± 1 ^g^	20.5 ± 1.5 ^de^	17 ± 1 ^e^	48 ± 2 ^d^
B16	62 ± 1 ^b^	76 ± 1 ^b^	51 ± 3 ^a^	38 ± 3 ^b^	95 ± 2 ^a^
Ellipticine	1.21 ± 0.02	0.9 ± 0.1	1.0 ± 0.1	1.10 ± 0.09	2.3 ± 0.2

Results are expressed as mean ± standard deviation. In each column, different letters correspond to significant differences (*p* < 0.05) between samples. GI_50_ values correspond to the extract concentration responsible for 50% of cell growth inhibition.

**Table 6:** Anti-inflammatory activity of the hydroethanolic extracts of *Cynara cardunculus* L. var *altilis* blades collected at different growth stages.

Sample	NO Production Inhibition (IC_50_, µg/mL)
B1	30 ± 3 ^ef^
B2	32 ± 2 ^e^
B3	53 ± 5 ^b^
B4	48 ± 2 ^c^
B5	39 ± 2 ^d^
B6	27 ± 1 ^f^
B7	72 ± 3 ^a^
B8	24.6 ± 0.5 ^g^
B9	32 ± 1 ^e^
B10	16.2 ± 0.4 ^g^
B11	13.5 ± 0.9 ^gh^
B12	12 ± 1 ^gh^
B13	10 ± 1 ^h^
B14	16 ± 1 ^g^
B15	30 ± 3 ^ef^
B16	56 ± 2 ^b^
Dexamethasone	16 ± 1

Results are expressed as mean ± standard deviation. In each column, different letters correspond to significant differences (*p* < 0.05) between samples. IC_50_ values correspond to the extract concentration needed to inhibit by 50% the nitric oxide (NO) production.

**Table 7:** Antibacterial activity of the hydroethanolic extracts of *Cynara cardunculus* L. var *altilis* blades collected at different growth stages.

Antibacterial Activity (mg/mL)												
Sample	*B. cereus*		*S. aureus*		*L. monocytogenes*		*E. cloacae*		*E. coli*		*S. typhimurium*	
	MIC	MBC	MIC	MBC	MIC	MBC	MIC	MBC	MIC	MBC	MIC	MBC
B1	0.58	1.17	1.17	2.33	1.17	2.33	1.17	2.33	1.17	2.33	1.17	2.33
B2	0.58	1.15	2.31	4.61	1.15	2.31	1.15	2.31	1.15	2.31	1.15	2.31
B3	1.17	2.33	2.33	4.66	0.58	1.17	2.33	4.66	1.17	2.33	2.33	4.66
B4	0.91	1.81	1.81	3.63	3.63	7.26	3.63	7.26	0.91	1.81	1.81	3.63
B5	0.86	1.72	1.72	3.43	1.72	3.43	1.72	3.43	0.86	1.72	1.72	3.43
B6	3.07	3.07	3.07	3.07	3.07	6.15	1.54	3.07	1.54	3.07	1.54	3.07
B7	3.57	3.57	0.89	0.89	1.78	3.57	1.78	3.57	1.78	3.57	1.78	3.57
B8	0.80	1.60	0.80	11.60	1.60	3.21	1.60	3.21	0.80	1.60	1.60	3.21
B9	1.61	1.61	0.81	1.61	0.81	3.22	0.81	1.61	0.81	1.61	0.81	1.61
B10	0.89	1.78	1.78	3.55	1.78	3.55	3.55	7.11	1.78	3.55	3.55	7.11
B11	0.78	1.55	1.55	3.10	1.55	3.10	1.55	3.10	1.55	3.10	1.55	3.10
B12	0.43	0.87	1.74	3.48	1.74	3.48	1.74	3.48	3.48	6.96	3.48	6.96
B13	0.77	1.54	0.77	1.54	0.77	3.07	0.77	3.07	0.77	3.07	0.77	3.07
B14	0.82	1.63	3.27	6.54	1.63	3.27	1.63	3.27	1.63	3.27	3.27	6.54
B15	0.58	1.16	2.32	4.64	2.32	4.64	2.32	4.64	2.32	4.64	1.16	2.32
B16	0.58	1.16	1.16	2.32	1.16	2.32	1.16	2.32	1.16	2.32	1.16	2.32
Streptomycin	0.10	0.20	0.04	0.10	0.20	0.30	0.20	0.30	0.20	0.30	0.20	0.30
Ampicillin	0.25	0.40	0.25	0.45	0.40	0.50	0.25	0.50	0.40	0.50	0.75	1.20

MIC: minimal inhibitory concentration; MBC: minimal bactericidal concentration.

**Table 8:** Antifungal activity of the hydroethanolic extracts of *Cynara cardunculus* L. var *altilis* blades collected at different growth stages.

Antifungal Activity (mg/mL)												
Sample	*A. fumigatus*		*A. versicolor*		*A. niger*		*P. funiculosum*		*P. ochrochloron*		*P. verrucosum* var. *cyclopium*	
	MIC	MFC	MIC	MFC	MIC	MFC	MIC	MFC	MIC	MFC	MIC	MFC
B1	1.83	3.66	1.83	3.66	0.92	1.83	1.83	3.66	1.83	3.66	0.92	1.83
B2	0.92	1.84	3.69	7.37	1.84	3.69	0.92	1.84	1.84	3.69	1.84	3.69
B3	1.86	3.71	1.86	3.71	0.93	1.86	1.86	3.71	0.93	1.86	1.86	3.71
B4	4.84	9.68	0.60	1.21	>9.68	>9.68	0.60	1.21	0.30	0.60	0.30	0.60
B5	4.58	9.16	1.14	2.29	>9.16	>9.16	1.14	2.29	0.57	1.14	0.57	1.14
B6	4.1	8.2	0.51	1.02	>8.2	>8.2	0.51	1.02	0.51	1.02	0.51	1.02
B7	1.19	2.38	1.19	2.38	1.19	2.38	0.59	1.19	0.59	1.19	1.19	2.38
B8	2.14	4.28	1.07	2.14	>8.56	>8.56	1.07	2.14	1.07	2.14	0.53	1.07
B9	1.07	2.15	1.07	2.15	2.15	4.3	1.07	2.15	1.07	2.15	1.07	2.15
B10	4.74	9.48	2.37	4.74	>9.48	>9.48	1.18	2.37	1.18	2.37	1.18	2.37
B11	0.78	1.55	0.39	0.78	0.78	1.55	0.39	0.78	0.78	1.55	0.78	1.55
B12	0.58	1.16	0.58	1.16	0.58	1.16	1.16	2.32	1.16	2.32	1.16	2.32
B13	2.05	4.1	1.02	2.05	1.02	2.05	1.02	2.05	0.51	1.02	1.02	2.05
B14	0.54	1.09	0.54	1.09	1.09	2.18	0.54	1.09	1.09	2.18	0.54	1.09
B15	3.64	7.28	0.91	1.82	0.91	1.82	1.82	3.64	1.82	3.64	0.91	1.82
B16	3.69	7.37	0.92	1.84	1.84	3.69	0.92	1.84	0.92	1.84	1.84	3.69
Ketoconazole	0.25	0.50	0.2	0.5	0.2	0.5	0.2	0.5	1.0	1.5	0.2	0.3
Bifonazole	0.15	0.20	0.1	0.2	0.15	0.2	0.2	0.25	0.2	0.25	0.1	0.2

MIC: minimal inhibitory concentration; MFC: minimal fungicidal concentration.

## Data Availability

Not applicable.
